# The City Blueprint Approach: Urban Water Management and Governance in Cities in the U.S.

**DOI:** 10.1007/s00267-017-0952-y

**Published:** 2017-11-03

**Authors:** Daniel Feingold, Stef Koop, Kees van Leeuwen

**Affiliations:** 1 Copernicus Institute for Sustainable Development and Innovation, Utrecht University, Heidelberglaan 2, Utrecht, 3584 CS The Netherlands; 2 KWR Watercycle Research Institute, Groningenhaven 7, Nieuwegein, 3433 PE The Netherlands

**Keywords:** City Blueprint, Water management, Capacity building, Water scarcity, Adaptive governance, Infrastructure deficit

## Abstract

In this paper, we assess the challenges of water, waste and climate change in six cities across the U.S.: New York City, Boston, Milwaukee, Phoenix, Portland and Los Angeles. We apply the City Blueprint^®^ Approach which consists of three indicator assessments: (1) the Trends and Pressures Framework (TPF), (2) the City Blueprint Framework (CBF) and (3) the water Governance Capacity Framework (GCF). The TPF summarizes the main social, environmental and financial pressures that may impede water management. The CBF provides an integrated overview of the management performances within the urban watercycle. Finally, the GCF provides a framework to identify key barriers and opportunities to develop governance capacity. The GCF has only been applied in NYC. Results show that all cities face pressures from heat risk. The management performances regarding resource efficiency and resource recovery from wastewater and solid waste show considerable room for improvement. Moreover, stormwater separation, infrastructure maintenance and green space require improvement in order to achieve a resilient urban watercycle. Finally, in New York City, the GCF results show that learning through smart monitoring, evaluation and cross-stakeholder learning is a limiting condition that needs to be addressed. We conclude that the City Blueprint Approach has large potential to assist cities in their strategic planning and exchange of knowledge, experiences and lessons. Because the methodology is well-structured, easy to understand, and concise, it may bridge the gap between science, policy and practice. It could therefore enable other cities to address their challenges of water, waste and climate change.

## Introduction

As centers of efficient infrastructure and services such as transport, communication, finance, energy, and water and sanitation, cities attract talent and skilled labor, which facilitates the exchange of ideas, knowledge development and boosts innovation (UN Habitat 2011a). Due in part to this attractiveness, the global urban population has skyrocketed from 746 million in 1950 to 3.9 billion in 2014 (United Nations 2014).By 2050, an additional 2.5 billion people are projected to reside in urban areas (United Nations 2014). The U.S. is a highly urbanized country with 80.7% of the American population residing in urban areas (U.S. Census Bureau 2012). Overall, cities in the U.S. are growing at a faster rate than the U.S. population. Between 2000 and 2013 the population in U.S. cities grew by 24.1 million, or 13.9 percent, while the total U.S. population grew 12.3% (Cohen et al. 2015). This population growth is expected to continue and by 2060, the U.S. population is projected to increase to 417 million people with 87 percent of the population living in urban areas (Colby and Ortman 2015).

At present, urban areas are the main drivers of global environmental change, as they constitute 75% of the global resource demand (Yeh and Huang 2012). Rapid population growth coupled with expected economic growth will lead to increased pressure on water resources. This is already evident from patterns of increasing groundwater depletion, saltwater intrusion, and pollution due to poor resource management (Hausmann et al. 2014; Bates et al. 2008; Vörösmarty et al. 2000). Globally, access to water has expanded, but progress on sanitation has been slower. Due to the enormous influence and impact, urban areas can act as both the cause and the solution to global environmental challenges and are key in achieving sustainable development (Yeh and Huang 2012; Koop and Van Leeuwen 2017). Recently, this has been highlighted too in the Atlas of Sustainable Development Goals of the World Bank (World Bank 2017).

Climate change will place even greater stress on both the urban environment and global water resources. The IPCC reports that heavy precipitation events are projected to become more frequent, which along with sea level rise will lead to increased flood risk, while the area affected by drought is likely to increase and water quality is likely to decrease (Bates et al. 2008). Furthermore, changes in seasonality as a result of earlier and decreased spring snowmelt will alter the timing of available water supplies and affect water infrastructure and industries that rely on established flows (Vaux 2015). Increasing water scarcity is already experienced in the western U.S., which is the region with the most urbanized and fastest growing population (Cohen et al. 2015). The Colorado River basin provides water for 33 million people in the West and experiences severe water scarcity for 5 months a year (EPA 2016; Hoekstra et al. 2012). In addition, an increase in flooding along the East Coast and Gulf Coast has been observed in recent years (Swee and Park 2014). In 2012, Hurricane Sandy exposed the vulnerability of New York City to extreme weather events, causing $19 billion in damages and lost economic activities while claiming the lives of 44 people (Goldstein et al. 2014). Even greater damage occurred when Hurricane Katrina devastated New Orleans in 2005. All these aspects have been addressed in detail in the third National Climate Assessment of the U.S. (Melillo et al. 2014).

Water infrastructure in developed countries is aging (OECD 2015a). The U.S. is facing an aging water infrastructure, a lack of government commitment, and insufficient financial support leading to an increasing investment deficit (Vaux 2015). The American Society of Civil Engineers reports that the water infrastructure of the U.S. is aging and degraded while funding for proper maintenance and replacement is lacking. Without increased funding there will be enormous impacts on public health and the economy (ASCE 2016).

**Fig. 1:**
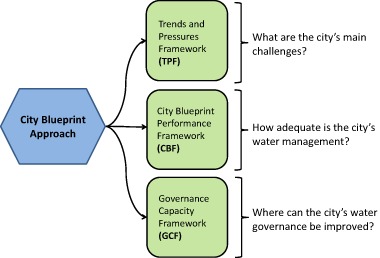
Overview of the City Blueprint Approach with three complementary assessment frameworks. The TPF and CBF are based on questionnaires, whereas the GCF is based on interviews

The City Blueprint Approach (Fig. 1) provides a platform in which cities can share their best practices and learn from each other (Koop and Van Leeuwen 2017). At present, 60 municipalities and regions in more than 30 countries have been assessed and best practices of these cities are summarized in a compendium (Koop et al. 2015). The Trends and Pressures Framework (TPF) and the City Blueprint Framework (CBF) provide cities with a quick and practical snapshot of their performance on water, waste and climate change (Koop and Van Leeuwen 2015a, 2015b). Furthermore, there has been a shift in traditional governance mechanisms in recent years as a response to environmental challenges and the reorganization of public, private and social sectors (Stoker 1998; Lockwood et al. 2010; Kersbergen and Waarden 2004; Romolini et al. 2016). This notion has also been addressed by international organizations (UN Water and Global Water Partnership 2007; UNDP 2013; OECD 2015b; 2016). Therefore, we recently developed an integrated empirically-based governance capacity framework (GCF) that enables consistent city comparisons and facilitates decision-making (Koop et al. 2017). The GCF facilitates good water governance by revealing areas of improvement for cities to increase their governance capacity (Fig. 1).

The goal of this study is (1) to understand the main similarities and differences in integrated water resources management (IWRM) in six cities in different regions of the U.S. (New York City, Boston, Milwaukee, Phoenix, Portland and Los Angeles), (2) to compare these assessments with resource efficient and adaptive cities in Europe and (3) to perform a GCF analysis of New York City to assess the key conditions, which determine its governance capacity, and based on these analyses (4) to map the gaps, opportunities and provide recommendations to address the urban challenges of water, waste, and climate change in the U.S.

## Methodology

### Selection of Cities in the U.S.

The research focuses on six cities in six different regions of the U.S. This selection of cities was based on our preference for rather big cities (population size), contacts in these cities, the accessibility of data, and the variations in their climate (e.g., temperature, precipitation, groundwater table) and geography (Melillo et al. 2014), and culture. The cities finally selected were New York, NYC (Mid-Atlantic), Boston, MA (New England), Milwaukee, WI (Great Lakes), Portland, OR (Pacific Northwest), Los Angeles, CA (Far West), and Phoenix, AZ (Southwest) shown in Table 1.

**Table 1:** General information about six cities in different regions of the U.S.

City	NYC	Boston	Milwaukee	Portland	Phoenix	Los Angeles
Population^a^	8,550,405	667,137	600,155	632,309	1,563,025	3,971,883
Daily Average Temperature (C°)^b^	12.5	10.8	8.8	12.5	23.9	18.6
Annual Average Rainfall (mm)^b^	1086	1112	883	914	204	379
Green Space (parks) (%)^c^	21.1	17	8.7	17.8	15	13.6
Groundwater Depletion 1900–2008 (Km^3^)^d^	0–3	N.A.	10–25	−10–0	50–150	3–10
Saltwater Intrusion^e^	Yes	Yes	No	No	No	Yes
Water Consumption (m^3^/person/y)^f^	173.815	81.51	128.5	132.5	255.2	156.22
Average Age of Sewer (y)^f^	84	100	45	80	50	50
Municipal Solid Waste Collected (Kg/cap/y)^f^	1637	1428	626	1550	535	842

^a^ U.S. Census Bureau (2015)

^b^ Arguez et al. (2010)

^c^ The Trust for Public Land (2015)

^d^ Konikow (2013)

^e^ EIP Water (2017a)

^f^ EIP Water (2017b)

New York City was selected for the GCF analysis because the city is a frontrunner in climate adaptation strategies (OneNYC 2017) and is the highest performing city in the governance category of the CBF (Koop and van Leeuwen 2015b). The city is a member of the C40 Cities Climate Leadership Group, a member of and one of the leading cities on climate change in the 100 Resilient Cities Network, as well as a signatory of the U.S. Conference of Mayors’ Climate Protection Agreement. Therefore, a governance capacity assessment of the city provides valuable insight into which governance conditions are most needed for developing the necessary governance capacity to implement a comprehensive climate adaptation strategy and address water challenges in other cities in the U.S.

### The City Blueprint Approach

The City Blueprint Approach (Fig. 1) consists of three complementary methodologies, (1) the TPF (EIP Water 2017a), (2) the CBF (EIP Water 2017b) and (3) the Water GCF (EIP Water 2017c) shown in Fig. 1. A detailed summary with all key references is provided in the E-Brochure (EIP Water 2017d). The methods were developed through a learning by doing approach. First, we developed a City Blueprint assessment based on 24 indicators (van Leeuwen et al. 2012; van Leeuwen 2013). Based on constructive feedback from cities we developed two separate indicator frameworks (Koop and van Leeuwen 2015a, 2015b) that embody the distinction between trends and pressures (TPF) and IWRM of a city (CBF). Recently the GCF was developed (Koop et al. 2017). Details about the methodologies are provided in the questionnaires and the publications provided in the E-Brochure (EIP Water 2017d).

#### Trends and pressures framework (TPF)

Every city has its own social, financial and environmental setting in which water managers have to operate. The TPF is developed to provide a concise understanding of these contextual trends and pressures. A distinction has been made between trends and pressures and IWRM performance (Koop and van Leeuwen 2015a). The TPF comprises of twelve indicators divided over social, environmental and financial categories (Table 2). Each indicator has been scaled from 0 to 4 points, where a higher score represents a higher urban pressure or concern. For seven indicators and sub-indicators we have proposed a scoring method as based on international quantitative standards such as the World Bank, World Health Organization and the Food and Agricultural Organization. The scores are determined using the ranking of the city amongst all available country scores. These scores are not normative and only provide an indication of the urban pressures with respect to global trends. Information on the scoring methods is provided in Koop and van Leeuwen (2015a), whereas very detailed information and examples are provided on our website (EIP Water 2017a). TPF scores are classified into five ordinal classes:0–0.5 points (no concern), 0.5–1.5 (little concern), 1.5–2.5 (medium concern), 2.5–3.5 (concern), and 3.5–4 (great concern). In the TPF, only indicators that are of concern or great concern (3 or 4 points) are explicitly communicated to the stakeholders. Further details on the data sources, calculation methods and scaling methods and limitations of the TPF are provided by Koop and Van Leeuwen (2015a). The application of these indicators is published in two other publications (Koop and Van Leeuwen 2015b; European Commission 2017).

**Table 2:** Basic method and features of the Trends and Pressures Framework and City Blueprint^®^ Framework (Koop and Van Leeuwen 2015a)

Trends and Pressures Framework (TPF)		
Goal	Baseline assessment of social, environmental and financial pressures	
Framework	Social pressures	1. Urbanization rate
		2. Burden of disease
		3. Education rate
		4. Political instability
	Environmental pressures	5. Flooding
		6. Water scarcity
		7. Water quality
		8. Heat risk
	Financial pressures	9. Economic pressure
		10. Unemployment rate
		11. Poverty rate
		12. Inflation rate
Data	Public data or data provided by the water and wastewater utilities	
Scores	0: no concern, 1: little concern, 2: medium concern, 3: concern and, 4: great concern	
Overall score	Trends and Pressures Index (TPI), the arithmetic mean of 12 indicators. Indicators scoring a concern or great concern (3 or 4 points) are communicated as a priority	
City Blueprint performance Framework (CBF)		
Goal	Baseline performance assessment of the state of IWRM	
Framework	Twenty-five indicators divided over seven broad categories:	
	1. Water quality	
	2. Solid waste	
	3. Basic water services	
	4. Wastewater treatment	
	5. Infrastructure	
	6. Climate robustness	
	7. Governance	
Data	Public data or data provided by the (water and wastewater utilities and cities based on a questionnaire (EIP Water 2017a)	
Scores	0 (low performance) to 10 (high performance)	
Overall score	Blue City Index^®^ (BCI), the geometric mean of 25 indicators	

#### City blueprint framework (CBF)

The CBF consists of 25 performance indicators that are scored from 0 (low performance) to 10 (high performance) and divided over seven broad categories covering the entire urban water cycle (Table 2). Detailed information about the data sources, calculation methods and scaling methods and limitations of the CBF are provided by Koop and Van Leeuwen (2015a) and the application of the methodology in many municipalities and regions have been published (Koop and Van Leeuwen 2015b; Gawlik et al. 2017). For these municipalities and regions a geometric mean of all 25 indicators, the Blue City Index (BCI), has been calculated. Further details on the data sources, calculation methods and scaling methods and limitations of the CBF are provided by Koop and Van Leeuwen (2015a). More details on data needs, calculations and examples are provided in the questionnaire on our website (EIP Water 2017b).

**Fig. 2:**
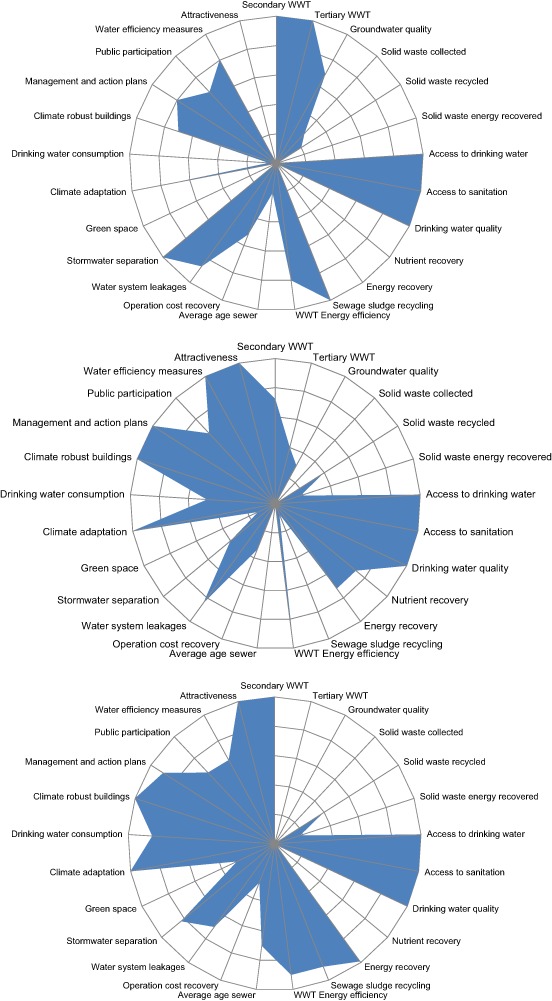
City Blueprints of Phoenix (top), New York City (center) and Boston (bottom), based on 25 performance indicators. The geometric mean of the indicators, i.e., the BCI scores, are 3.9, 4.8, and 5.4, respectively

A hierarchical clustering analyses of the 25 indicator scores of many municipalities and regions enabled the development of an empirical-based categorization of consecutive levels of IWRM worldwide (Table 3; Koop and van Leeuwen 2015b). In this paper we compare the result of six cities in the U.S. with the six cities with the highest BCI of a total of 60 municipalities and regions in order to provide recommendations on improvements. The cities with the highest BCI are categorized as resource efficient and adaptive cities (Table 3). The top 6 cities in the resource efficient and adaptive cities category were Amsterdam and Groningen (the Netherlands) and Helsingborg, Malmo, Kristianstad and Stockholm (Sweden) as shown in Fig. 2.

**Table 3:** Categorization of IWRM performance based on a cluster analysis of 25 City Blueprint indicators of municipalities and regions (Koop and Van Leeuwen 2015b)

BCI	Categories of IWRM in cities
0–2	Cities lacking basic water services.
	Access to potable drinking water of sufficient quality and access to sanitation facilities are insufficient. Typically, water pollution is high due to a lack of wastewater treatment (WWT). Solid waste production is relatively low but is only partially collected and, if collected, almost exclusively put in landfills. Water consumption is low, but water system leakages are high due to serious infrastructure investment deficits. Basic water services cannot be expanded or improved due to rapid urbanization. Improvements are hindered due to insufficient governance capacity and funding gaps
2–4	Wasteful cities.
	Basic water services are largely met but flood risk can be high and WWT is insufficiently covered. Often, only primary and a small portion of secondary WWT is applied, leading to large-scale pollution. Water consumption and infrastructure leakages are high due to the a lack of environmental awareness and infrastructure maintenance. Solid waste production is high, and waste is almost completely dumped in landfills. In many cases, community involvement is relatively low
4–6	Water efficient cities
	Cities are implementing centralized, well-known, technological solutions to increase water efficiency and to control pollution. Secondary WWT coverage is high, and tertiary WWT is rising. Water-efficient technologies are partially applied, infrastructure leakages are substantially reduced but water consumption is still high. Energy recovery from WWT is relatively high, while nutrient recovery is limited. Both solid waste recycling and energy recovery are partially applied. These cities are often vulnerable to climate change, e.g., urban heat islands and drainage flooding, due to poor adaptation strategies, limited storm water separation and low green surface ratios. Governance community involvement has improved
6–8	Resource efficient and adaptive cities
	WWT techniques to recover energy and nutrients are often applied. Solid waste recycling and energy recovery are largely covered, whereas solid waste production has not yet been reduced. Water-efficient techniques are widely applied, and water consumption has been reduced. Climate adaptation in urban planning is applied, e.g., incorporation of green infrastructures and storm water separation. Integrative, (de)centralized and decentralized as well as long-term planning, community involvement, and sustainability initiatives are established to cope with limited resources and climate change
8–10	Water wise cities
	There is no BCI score that is within this category so far. These cities apply full resource and energy recovery in their WWT and solid waste treatment, fully integrate water into urban planning, have multi-functional and adaptive infrastructures, and local communities promote sustainable integrated decision-making and behavior. Cities are largely water self-sufficient, attractive, innovative and circular by applying multiple centralized and decentralized solutions

#### Governance capacity framework (GCF)

The GCF has been developed to address governance, which is a crucial factor in the sustainability of cities (OECD 2015b; 2016; Koop and Van Leeuwen 2017). This was the reason why we developed a comprehensive framework for cities that can (1) compare cities and provide a better empirical-based understanding of the key enabling governance conditions, and (2) reveal the limiting conditions in order to formulate pathways for an effective and efficient improvement in the local capacity to govern water challenges. Altogether, urban areas face five main urban water challenges that will increase in relevance due to continued urbanization and climate change. These challenges are: (1) water scarcity, (2) flood risk, (3) wastewater treatment, (4) solid waste treatment, and (5) urban heat islands (Koop et al. 2017).

The GCF comprises nine governance conditions, each with three indicators. For each of the twenty-seven indicators, a Likert-type scoring scale has been developed that ranges from very encouraging (++) to very limiting (--). The GCF has been further operationalized by developing specific questions linked with Likert-type scoring and has recently been applied for the city of Amsterdam (Koop et al. 2017), Quito (Schreurs et al. 2017) and Ahmedabad (Aartsen et al. 2017). A detailed description of the GCF methodology, each indicator, the scoring methodology, and its limitations are provided by Koop et al. (2017). The methodology is publicly available in order to ensure full transparency (EIP Water 2017c). An overview of the GCF method as well as the results are presented in the results section below.

### Data Gathering

The data for the TPF and CBF were gathered in two successive steps. First, an extensive literature study was carried out to determine the preliminary scores for all 43 TPF and CBF indicators and sub-indicators. These preliminary scores were presented to the authorities in the cities. Key persons within these organizations were asked to provide feedback.

The data for the GCF for New York City were gathered by conducting fifteen qualitative semi-structured interviews. Eight interviews were held with respondents that work for the state or city government. Seven interviews were held with respondents that work for non-governmental organizations (NGOs) that are influential in New York City water governance. The relevant stakeholders were identified and a number of stakeholders were interviewed based on availability and willingness to participate. Subsequently, the snowball method was employed in order to facilitate efficient navigation of the New York City water governance network and identify other available relevant stakeholders in the network. NGOs were included in order to obtain multiple viewpoints from stakeholders as it was determined that solutions to complex environmental problems need to include stakeholder participation in decision making (Bäckstrand 2003; Bingham et al. 2005).

The organizations involved in this study were the NYC Department of Environmental Protection, the Mayor’s Office of Resiliency and Recovery, the NYC Department of Sanitation, the Waterfront Alliance, the Science and Resiliency Institute at Jamaica Bay, the Natural Resources Defense Council, GrowNYC, Riverkeeper, NYC Department of Parks and Recreation, West Harlem Environmental Action (WE ACT), NYC H2O, and the New York Soil and Water Conservation District.

### Other Municipalities and Regions

**Fig. 3:**
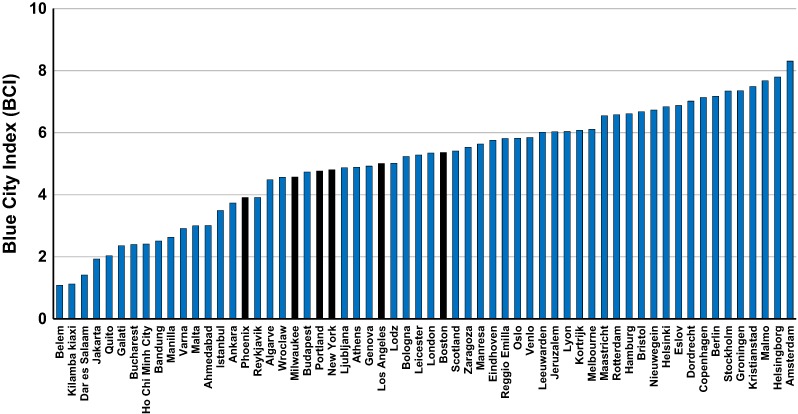
The Blue City Index of 60 municipalities and regions in more than 30 different countries. BCI values of cities in the U.S. are highlighted in black

Assessments of the TPF and CBF in many other municipalities and regions have been published previously (Koop and Van Leeuwen 2015b; 2017; Gawlik et al. 2017). In this paper we have included another 15 cities allowing for a better comparison with cities in the U.S., i.e., Bristol and Leicester (both UK), Leeuwarden and Groningen (both in the Netherlands), Ahmedabad (India), Kortrijk (Belgium), Quito (Ecuador), Jakarta and Bandung (Indonesia), Manila (Philippines) and another five cities in the U.S. (Fig. 3). At this point, there is a strong bias towards cities in Europe as only 19 non-European cities have been assessed so far, i.e., Ankara and Istanbul (Turkey), Jerusalem (Israel), Kilamba Kiaxi (Angola), Dar es Salaam (Tanzania), Ahmedabad (India), Ho Chi Minh City (Vietnam), Bandung and Jakarta (Indonesia), Manila (Philippines), Melbourne (Australia), Belém (Brazil), Quito (Ecuador), and the six U.S. cities. Therefore, we emphasize the strong necessity of extending our work to cities outside Europe.

## Results

### Trends and Pressures

For all TPF indicators, the scores varied from no to medium concern. There were three exceptions: (1) heat risk is a great concern for Phoenix and Los Angeles, a concern for Milwaukee, New York City and Boston, and of little concern for Portland; (2) saltwater intrusion is a concern for New York City, Boston and Los Angeles (Table 1); (3) urban drainage flooding are great concerns both for New York City and Boston.

### City Blueprints

The CBF provides a snapshot of each city’s water management performance. Examples of the City Blueprint of Phoenix, New York City and Boston are shown in Fig. 2. The six cities in the U.S. score rather well on all City Blueprint indicators, but there are also options for improvement. The options are provided by the City Blueprint indicators for which rather low scores are observed, i.e., tertiary waste water treatment, solid waste collection/generation, energy recovery from solid waste, nutrient recovery from waste water, average age of the sewer (maintenance of underground infrastructure) and green space. The results are shown in Fig. 3 next to all other cities and regions assessed so far. Based on the overall performance, the cities can be categorized based on the BCI scores assigned to each (Table 3). Portland, Milwaukee, Los Angeles, New York City and Boston, with BCIs between 4 and 6, are categorized as water efficient cities while Phoenix (BCI 3.9) is categorized as a wasteful city.

**Fig. 4:**
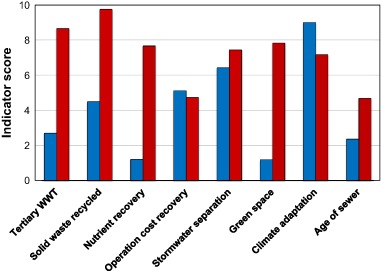
A comparison of average scores for eight City Blueprint indicators for six U.S. cities (left; blue bars) and six cities with the highest BCI scores (right; red bars) as shown in Fig. 3

The results show that all six U.S. cities score high in basic water services and secondary wastewater treatment while Phoenix is the only U.S. city to score high on tertiary wastewater treatment. All U.S. cities score high on climate adaptation due to the implementation of publicly available local climate adaptation plans but low on green space and stormwater separation, which increases vulnerability to climate change. Figure 4 clearly provides options where U.S. cities can improve compared to other cities. The average scores for operation cost recovery in the U.S. is 5.1 and hardly differs from the average score of 4.7 for the six cities with the highest BCI (Fig. 4). The same is true for the average water system leakages of 13.2 and 11.3%, respectively.

### The Water Governance Capacity of New York City

Table 4 shows the detailed results of the governance capacity assessment for the five identified urban water challenges: (1) water scarcity, (2) flood risk, (3) wastewater treatment, (4) solid waste treatment, and (5) urban heat islands.

**Fig. 5:**
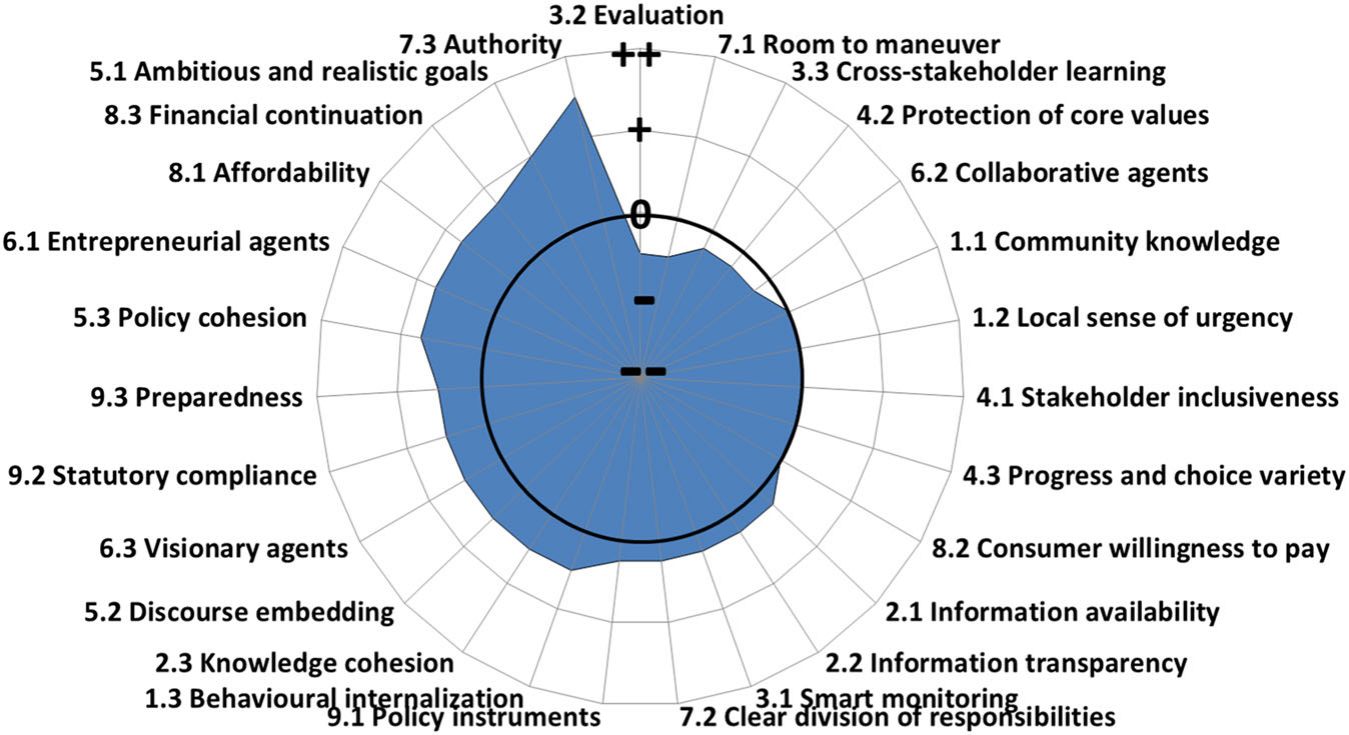
Results of the GCF analysis. Limiting GCF indicators, with scores below zero, are 3.2 evaluation, 3.3 cross-stakeholder learning, 4.2 protection of core values, 6.2 collaborative agents, and 7.1 room to maneuver

Figure 5 shows the average score for each of the five water challenges for all 27 indicators. The governance capacities to respectively address flood risk, wastewater treatment and solid waste treatment are relatively well developed. The governance capacity for water scarcity is slightly lower with a few indicators that have a limiting effect, whereas the development of capacity to govern the challenge of urban heat islands can be considered a priority (Table 4). In particular, five indicators are found to be limiting the overall governance capacity for almost all water challenges (Fig. 5):

- 3.2 *Evaluation*: Current policy and implementation are in many cases insufficiently assessed and improved throughout the decision-making and implementation process. Moreover, there is room to improve the quality of existing evaluation methods, the frequency of their application, and the level of learning (EIP water 2017c).
- 3.3 *Cross-stakeholder learning*: Stakeholders have only limited opportunity to interact with other stakeholders and the engagement is relatively superficial decreasing opportunities to learn from each other (EIP water 2017c).
- 4.2 *Protection of core values*: There are risks that engaged stakeholders do not feel confident that their core values (e.g., flood safety of their property) are being protected in the stakeholder engagement process. Sometimes commitment to early end-results are being demanded, opportunities for active involvement or knowledge coproduction are low, and exit procedures are clear and transparent (Ridder et al. 2005). These components all may limit a stakeholder engagement process that ensures protection of core values of all engaged stakeholders (EIP water 2017c).
- 6.2 *Collaborative agents*: In order to drive change, agents of change are required to show direction, motivate others to follow and mobilize the resources required (EIP water 2017c).
- 7.1 *Room to maneuver*: The freedom and opportunity to develop a variety of alternatives, approaches, and to form new partnerships that can adequately address existing or emerging issues can be improved considerably (EIP water 2017c).

**Table 4:** Outcome of the water governance capacity framework (GCF) analysis of New York City. The governance capacity scores to address each challenge range from very encouraging (++) to very limiting (−−)

Dimension	Conditions	Indicators	Water scarcity	Flood risk	Waste water treatment	Solid waste treatment	Urban heat islands
Knowing	1. Awareness	1.1 Community knowledge	0	0	0	0	0
		1.2 Local sense of urgency	−	0	0	+	0
		1.3 Behavioral internalization	+	+	0	+	0
	2. Useful knowledge	2.1 Information availability	0	0	+	0	0
		2.2 Information transparency	0	0	0	0	+
		2.3 Knowledge cohesion	+	+	0	0	+
	3. Continuous learning	3.1 Smart monitoring	++	0	+	0	−−
		3.2 Evaluation	0	0	0	0	−−
		3.3 Cross-stakeholder learning	−	0	+	0	−
Wanting	4. Stakeholder engagement process	4.1 Stakeholder inclusiveness	0	+	+	0	−
		4.2 Protection of core values	0	0	0	0	−
		4.3 Progress and variety of options	0	+	0	0	0
	5. Management ambition	5.1 Ambitious realistic management	++	+	0	+	+
		5.2 Discourse embedding	0	+	+	0	+
		5.3 Management cohesion	+	+	0	+	+
	6. Agents of change	6.1 Entrepreneurial agents	0	+	+	+	+
		6.2 Collaborative agents	−	0	0	0	0
		6.3 Visionary agents	0	++	0	++	0
Enabling	7. Multi-level network potential	7.1 Room to maneuver	0	+	0	−	−
		7.2 Clear division of responsibilities	0	+	+	0	0
		7.3 Authority	++	+	++	+	+
	8. Financial viability	8.1 Affordability	+	+	+	+	+
		8.2 Consumer willingness to pay	0	+	0	0	0
		8.3 Financial continuation	+	+	+	+	0
	9. Implementing capacity	9.1 Policy instruments	0	+	0	0	+
		9.2 Statutory compliance	0	+	0	+	+
		9.3 Preparedness	+	+	0	+	0

Our study shows that in particular indicators related to continuous learning appear to be limiting the overall governance capacity (Table 4). Continuous learning (Folke et al. 2005) and social learning are essential in the iterative process of governing, improving and reassessing current policy and implementation. The level of learning is operationalized by the three loops of social learning (e.g., Pahl-Wostl 2009) ranging from refining current management, critical investigation of assumptions, and questioning fundamental beliefs, norms and values (Koop et al. 2017).

## Discussion

### Comparison With Top Performing Cities

In this paper, we compared the IWRM performance in cities in the U.S. with the top six cities categorized as resource efficient and adaptive cities, in order to gain an integrated understanding of practices that can be improved to become resource efficient and adaptive cities (Table 3 and Fig. 4). When compared to these cities, cities in the U.S. receive relatively low scores in the solid waste treatment category, mainly due to the large amount of solid waste produced by U.S. households and the low percentage of solid waste that is recycled or incinerated with energy recovery. In addition, the cities score low on wastewater nutrient recovery with only two cities (New York and Boston) employing any nutrient recovery.

According to the OECD (2015c), the U.S. tops the list of countries on solid waste generation (735 kg/capita/year). Resource recovery (recycling and composting) for the U.S. is 35%, incineration (with energy recovery) is 12 % and landfilling is 54%. Freshwater abstraction per capita (1580 m^3^/capita/year) is also very high in the U.S. and the same is true for drinking water use. According to the UNDP (2006) drinking water use in the U.S. has been estimated at 575 liters per capita per day (210 m^3^/capita/year). Similar observations have been made in our City Blueprint analyses for cities in the U.S. Based on this information, as well as the information provided in Figs. 3 and 4, the following options for improvement are available for cities in the U.S.: reductions in solid waste production and drinking water consumption, as well as further improvements in the areas of tertiary wastewater treatment, solid waste recycling, nutrient recovery from wastewater, stormwater separation and green space, as part of the broader topic of urban land use planning. Furthermore, underground pipelines are among the most valuable, yet neglected assets in the public arena as they provide essential services such as the supply of drinking water and collection of wastewater (UNEP 2013; OECD 2015a). Therefore, improved maintenance of sewer systems and drinking water distribution networks is very relevant too. The water infrastructure refurbishment deficit, also poses feasible opportunities to redesign the infrastructure with more stormwater storage possibilities, rainwater recycling, infiltration elements, and separation of stormwater and wastewater pipelines. This can strongly reduce flood damage, alleviate water demands, reduce water pollution, and contribute to a more attractive neighborhood.

### The Challenge of Resilient Water Infrastructures

Responsible for more than 70% of global energy-related carbon dioxide emissions, cities represent the single greatest opportunity for tackling climate change as well as mitigation and adaptation (UN Habitat 2011b; Koop and Van Leeuwen 2017). They also radically alter land use, ecosystems and hydrological systems (Krause 2011; Grimm et al. 2008; Yeh and Huang 2012). However, cities also hold the key for solving global environmental problems as they have the authority over policy on transportation, land-use, building codes, electricity production and transmission, waste management, and IWRM (Yeh and Huang 2012; Krause 2011; Koop and Van Leeuwen 2017).

Cities in OECD countries have not solved water management issues (OECD 2015a; 2016). While they currently enjoy relatively high levels of protection against water risks, they face disquieting challenges, including the proven difficulty of upgrading and renewing existing infrastructures, and heightened uncertainty about future freshwater availability and quality. According to the OECD (2015a), these cities are entering a new era, characterized by the need to retrofit existing assets into more adaptable infrastructure, by different combinations of financing tools and by new roles for stakeholders in water management.

Cities need to protect their citizens against water-related disasters (e.g., droughts and floods), to guarantee freshwater availability and high-quality groundwater, surface water and drinking water. Cities also need to have adequate infrastructure in response to climate, demographic and economic trends and pressures (OECD 2015a). The cost of urban infrastructure is high. In our recent review (Koop and Van Leeuwen 2017), we referred to the estimates published by UNEP (2013). For the period 2005–2030 about US$ 41 trillion is needed to refurbish the old (in mainly developed countries) and build new (mainly in the developing countries) urban infrastructures. The cost of water infrastructure (US$ 22.6 trillion) is estimated at more than that for energy, roads, rail, air and seaports put together. According to UNEP (2013) the wastewater infrastructure is responsible for the largest share of this 22.6 trillion.

In the absence of a federal initiative on climate change, city governments have become the leaders of U.S. climate protection efforts (Krause 2011). Similarly, federal support for water infrastructure is lacking and funding for water infrastructure has decreased in real purchase power since the mid-1980’s and state and city governments now account for 96% of all spending on water and wastewater infrastructure (Eskaf 2015). As a result, U.S. cities have the responsibility and opportunity to manage their water resources sustainably.

At present, it is estimated that there are 240,000 water main breaks and 75,000 sewer overflows that discharge 3 to 10 billion gallons of untreated wastewater every year across the nation (Mehan 2002). The United States Environmental Protection Agency has identified a potential $500 billion gap in funding for the nation’s drinking and wastewater infrastructure by 2020 (Mehan 2002). The costs of treating and delivering drinking water exceed the available funds needed to sustain the systems (Vaux 2015). However, elected politicians are unwilling to allocate funds to replace and maintain the water infrastructure and consumer costs for water supply and wastewater treatment, on average 0.3% of disposable income, only offset a small part of the required expenses (Pincetl et al. 2016; Vaux 2015). This may have consequences for the financial continuation of water services in the city. These findings are consistent with the CBF assessment of the six U.S. cities as well as the GCF assessment of New York City.

### Flooding

Sea level rise, storm surge, and heavy downpours, in combination with the pattern of continued development in coastal areas, are increasing damage to U.S. infrastructure including roads, buildings, and industry (Wahl et al. 2015). Flooding along rivers, lakes, and in cities following heavy downpours, prolonged rains, and rapid melting of snowpack is exceeding the limits of flood protection infrastructure designed for historical conditions (Melillo et al. 2014). Overall, the low percentage of green area and the relatively low percentage of stormwater separation in U.S. cities increases the impact of heavy precipitation and flooding events. Similar observations have been made by Leonardsen (2017) in her recent study on climate change adaptation solutions in U.S. cities. In New York City it was found that the governance capacity score for flood risk is the most encouraging out of all of the challenges. This may be a reaction to Hurricane Sandy in 2012, which resulted in increased funding, political attention and a common vision (Cohn 2016). However, the GCF assessment (Table 4) finds that 1.1 community knowledge is low, illustrating that people may have begun to slip back to business as usual, although New York City recently provided impressive long-term integrated plans to meet their current and future challenges (OneNYC 2017). Such long-term integrated plans are needed and may also save residents millions of dollars. This is fully in line with our first recommendation to cities, i.e., “cities require a long-term framing of their sectoral challenges into a proactive and coherent Urban Agenda to maximize the co-benefits and to minimize their cost” (Koop and Van Leeuwen 2017).

### Water Scarcity

Groundwater is the world’s largest accessible source of fresh water. It plays a vital role in satisfying basic needs for agricultural and industrial activities and drinking water. Water scarcity is occurring in the western U.S. (Hoekstra et al. 2012; Wada et al. 2012; Vaux 2015; de Graaf et al. 2015), and the extreme drought in California can be viewed as a lesson on managing water in a warmer, more densely populated world (Melillo et al. 2014; AghaKouchak et al. 2015). Hoekstra et al. (2012) estimate that agriculture accounts for 92 % of the global blue water footprint. Drinking water only represents a very small proportion of global freshwater use.

Drinking water consumption is high for all of the U.S. cities with the exception of Boston (Fig. 2). When addressing water scarcity in New York City governance indicators that were limiting were indicator 1.2 local sense of urgency, indicator 3.3 cross-stakeholder learning, and indicator 6.2 collaborative agents. However, water scarcity is not a pressing issue for New York City while it is a tremendous challenge for Phoenix and Los Angeles. Through a literature review it was found that water management in the West is managed by a maze of water agencies with unclear and conflicting goals (Lyon 2009). Public education is important for addressing water scarcity as water use declines when users know the source of their water is limited and understand how to reduce their consumption (Vaux 2015). In addition, wastewater recycling and the use of tertiary wastewater treatment is high in Phoenix but can continue to be increased in Los Angeles while rationing and the inclusion of a scarcity value in the price of water can encourage further conservation (Vaux 2015).

### Urban Water Challenges are Water Governance Challenges

Governance is the biggest obstacle for the sustainable management of water resources and “water crises are primarily governance crises” (Pahl-Wostl 2009; OECD 2015b). Interviews for the governance capacity analysis of New York City were held in summer 2016 and a summary of the results is shown in Fig. 5. There were concerns about the hierarchical structure of New York City’s water governance network. The network is dominated by city agencies (NYCDEP, DSNY, ORR, NYC Parks), which have strong authority (indicator 7.3). While these agencies emphasize stakeholder meetings, cross-stakeholder learning (indicator 3.3) is limited, which results in restricted collaboration (indicator 6.2) and a feeling that stakeholders are underrepresented in the end-results (indicator 4.2). This leads to a deterioration of trust between stakeholders and limits the room to maneuver (indicator 7.1) of stakeholders to develop and communicate alternatives. Furthermore, evaluations (indicator 3.2) are oftentimes performed internally, and lack comprehensiveness while focusing on outputs rather than outcomes. Overall, these limiting governance indicators can hinder New York City’s capacity to undergo effective change and enhance preparedness for uncertain futures.

In order to improve New York City’s urban water governance capacity focus should be placed on developing trust relations. Recently, the City has taken a big step in the right direction. The Mayor’s Office unveiled One New York: The Plan for a Strong and Just City (OneNYC), which addresses the city’s challenges through a long-term, integrated approach (OneNYC 2017). The plan was created with extensive community engagement and the goal of increasing civic engagement is central to many of its initiatives. The inclusion of a diverse group of stakeholders in the formulation of OneNYC is important in rebuilding trust with the community. In the future, this newfound trust can serve to open a dialog between various stakeholders and city agencies and result in new fit-for-purpose partnerships to successfully address unconventional challenges and build a more resilient city.

## Implications and Future Directions

The results presented in this paper support the conclusions of Vaux (2015) for the U.S. “The picture of urban water management—current and future—that emerges for the United States is characterized by the water paradox of developed countries. Virtually the entire population of the country has access to healthful water supplies and fully adequate sanitation services. Yet, urban residents and water managers are faced with an array of future water management problems that appear to be just as daunting as those faced by countries which are not fully served*.*” Similar observations have been made by the OECD for all developed countries (OECD 2015a). According to the Third U.S. National Climate Assessment (Melillo et al. 2014), climate change may worsen water services in the U.S. This may also affect the quality of life, particularly in cities (Koop and Van Leeuwen 2017).

Our research facilitates the practical application of IWRM to the city level by identifying the trends and pressures, the current IWRM performances and the governance capacity of a city when addressing water scarcity, flood risks, wastewater treatment, solid waste treatment, and urban heat islands. Cities are needed to enhance city-to-city learning and to improve governance capacities necessary to accelerate effective and efficient transitions (Koop and Van Leeuwen 2017).

The City Blueprint Approach provides a quick assessment of the challenges of water, waste and climate change in cities. Adaptive and anticipatory water management approaches should be embraced as cities throughout the world continue to grow and face ever increasing and complex water challenges as well as the uncertain consequences of climate change (Romolini et al. 2016; OECD 2015a; 2016). In order to facilitate the adoption of these approaches governance capacity must be strengthened (OECD 2015b; Koop and Van Leeuwen 2017). A better understanding of citywide environmental challenges and governance networks can inform evaluations of their effectiveness, contributing to improved environmental management by reducing costs and improving overall effectiveness through the exploration of win-win’s (Koop and Van Leeuwen 2017; Romolini et al. 2016). The following recommendations are suggested:

- 1. Based on the trends and pressures analyses, heat risk is a major concern or concern for five out of the six U.S. cities in this study and can be better addressed through the creation of specific plans to tackle urban heat island effects, which include monitoring and evaluation in order to determine the most effective city-specific measures. Saltwater intrusion is a concern for New York City, Boston and Los Angeles and urban drainage flooding are great concerns both for New York City and Boston.
- 2. Based on the City Blueprint analyses, long-term strategic planning and increased capital investments are needed to improve tertiary wastewater treatment, solid waste recycling, nutrient recovery from wastewater treatment, storm water separation, and infrastructure maintenance and improvement in U.S. cities (Figs. 2 and 3).
- 3. The current political emphasis on improving U.S. infrastructure should not be limited to aboveground infrastructure. Water infrastructure (drinking water networks, sewers and sewage treatment plants) and green space in cities are major challenges in the U.S. (Fig. 4). In fact, multi-functional land use and multi-functional infrastructure should be explored further.
- 4. Urban land use planning, supported by well-planned and well-managed initiatives and investments, can help address these challenges. One of these components is the need to increase green space to enhance the resiliency of cities to more frequent and intense flooding and heat waves in addition to its overall benefits on human wellbeing and the economy (Fig. 4).
- 5. Based on the governance capacity analysis of NYC, monitoring and evaluation of projects and improved cross-stakeholder learning through e.g., workshops, which engage different levels of management is proposed to increase continuous learning and make water governance more effective (Fig. 5).
